# Characterization of a Potent and Orally Bioavailable Lys-Covalent Inhibitor of Apoptosis Protein (IAP) Antagonist

**DOI:** 10.1021/acs.jmedchem.3c00467

**Published:** 2023-06-01

**Authors:** Parima Udompholkul, Ana Garza-Granados, Giulia Alboreggia, Carlo Baggio, Jack McGuire, Scott D. Pegan, Maurizio Pellecchia

**Affiliations:** Division of Biomedical Sciences, School of Medicine, University of California, Riverside, 900 University Avenue, Riverside, California 92521, United States

## Abstract

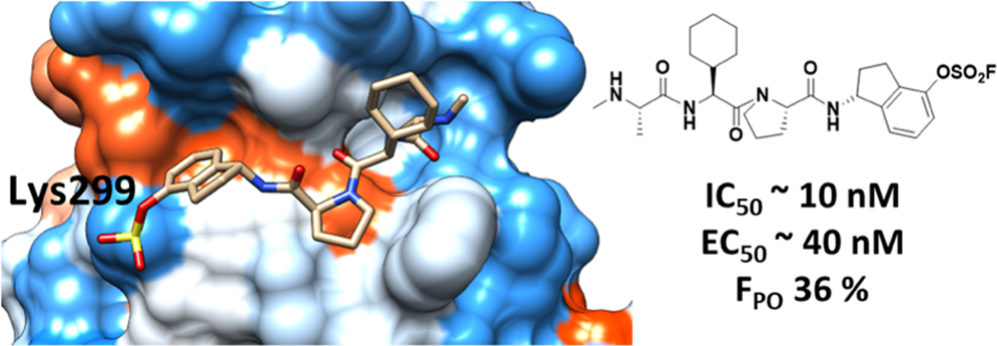

We have recently reported on the use of aryl-fluorosulfates in designing water- and plasma-stable agents that covalently target Lys, Tyr, or His residues in the BIR3 domain of the inhibitor of the apoptosis protein (IAP) family. Here, we report further structural, cellular, and pharmacological characterizations of this agent, including the high-resolution structure of the complex between the Lys-covalent agent and its target, the BIR3 domain of X-linked IAP (XIAP). We also compared the cellular efficacy of the agent in two-dimensional (2D) and three-dimensional (3D) cell cultures, side by side with the clinical candidate reversible IAP inhibitor LCL161. Finally, *in vivo* pharmacokinetic studies indicated that the agent was long-lived and orally bioavailable. Collectively our data further corroborate that aryl-fluorosulfates, when incorporated correctly in a ligand, can result in Lys-covalent agents with pharmacodynamic and pharmacokinetic properties that warrant their use in the design of pharmacological probes or even therapeutics.

## Introduction

In recent years, we have witnessed a resurgence of targeted covalent inhibitors in oncology research^1−14^ that translated in the Food and Drug Administration (FDA) approval of several acrylamide-based Cys-covalent inhibitors, including Sotorasib (Lumakras), Osimertinib (Tagrisso), Ibrutinib (Imbruvica), Neratinib (Nerlynx), and Afatinib (Gilotrif), to cite a few. The clinical success of these agents can be most likely attributed to their potency, and the sustained inhibition of their targets, being irreversible, both representing critically important pharmacodynamic and pharmacokinetic advantages over reversible inhibitors. However, while the introduction of acrylamides provides a proper balance of Cys-reactivity and drug stability *in vivo*, the approach is limited to those targets that present a Cys in proximity to their binding sites. Currently, increased drug discovery efforts focus on the Cysteinome,^15−20^ which is the target space, albeit limited, that contains a druggable Cys residue. For example, of the several activating mutations of KRAS, driving malignant transformation in several solid tumors, only the KRAS(G12C) could be targeted with this approach leading to recently approved covalent drugs for this specific mutation.^21−23^ Hence, we and others have been exploring the possibility to target other more frequently occurring nucleophilic amino acids such as, Lys, Tyr, or His, and probed a variety of targeting electrophiles^24−33^ comparing the reactivity of aryl-sulfonyl fluoride^10,27,34,35^ and aryl-fluorosulfate^26,36−39^ warheads when inserted in binding peptides targeting the inhibitors of apoptosis proteins (IAPs).^35,39−42^ Our recent work suggested that certain aryl-fluorosulfates, when properly juxtaposed to nucleophilic Lys residues, could provide the proper balance of Lys-reactivity, stability, and cell permeability, that resembled the properties of acrylamides when targeting Cys residues.^39^ The approach seems particularly advantageous to derive potent Lys-covalent inhibitors of protein–protein interactions (PPIs) for therapeutic use,^24,25^ given that obtaining potent and drug-like inhibitors of PPIs remains a challenging task.

Using a variety of biophysical^43^ and structure-based approaches,^24,25,35,39^ we designed and tested several Lys-covalent agents based on the tetrapeptide of sequence Ala-Val-Pro-Phe (AVPF) that interacted with various members of the IAP family, including X-linked IAP (XIAP), cellular inhibitor of apoptosis protein 1 (cIAP1), and cellular inhibitor of apoptosis protein 2 (cIAP2).^44−47^ Reversible AVPF mimetics were developed as potential therapeutic agents,^48−66^ including the clinical candidate LCL161^67−71^ (Table 1). We recently reported on compound **142D6** (Table 1) as a potent aryl-fluorosulfate-based Lys-covalent pan-IAP agent.^25^ The agent was designed to juxtapose more directly an aryl-fluorosulfate with a Lys residue within the binding pocket of the BIR3 domain of XIAP. Here, we report on further biochemical and biophysical characterizations of this agent, including the X-ray structure of the covalent complex with the BIR3 domain of XIAP that unambiguously identified the targeted Lys residue. Moreover, cellular assays in two-dimensional (2D) and three-dimensional (3D) cultures with breast cancer cells, and *in vivo* pharmacokinetics data further validated the use of these agents as innovative pharmacological tools. The data suggest that aryl-fluorosulfates represent valuable electrophiles for the development of pharmacologically viable covalent chemical probes or even therapeutics.

**Table 1:**
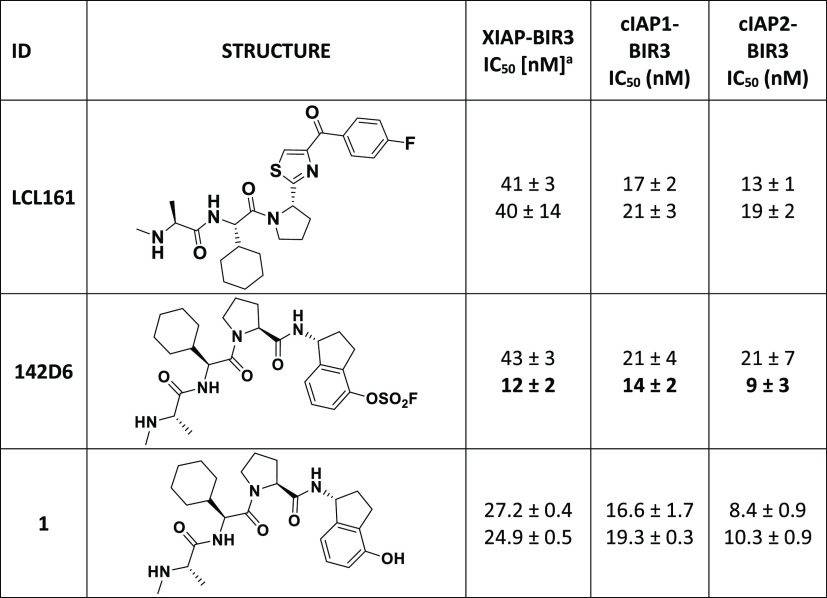
Structure and DELFIA Displacement IC_50_ Values for the Compounds under Investigation^a^

a IC_50_ values calculated from dose–response curves obtained without preincubation (top values) and after 6 h preincubation (bottom values) of protein and test ligands at room temperature. Incubation time dependence of covalent agents **142D6** is evident by the reduced IC_50_ values when the displacement assay was conducted with 6 h preincubation (bolded values). Data are presented as mean ± standard error (SE) of at least two replicate measurements.

## Results and Discussion

### *In Vitro* Biophysical, Biochemical, and Structural Characterization of **142D6** Interactions with the BIR3 Domain of XIAP

To quantify the ability of **142D6** to covalently target the BIR3 domains of XIAP, cIAP1, and cIAP2, we tested it side by side with clinical candidate LCL161 in a variety of biochemical and biophysical assays *in vitro*. First, we used a dissociation-enhanced lanthanide fluorescence immunoassay (DELFIA) displacement assay as described previously^43^ that measures the ability of test agents to compete for the binding of a reference biotinylated AVPI peptide. IC_50_ values were obtained from dose–response curves measured with or without 6 h preincubation of the test ligand and the protein domains (Table 1).

**Figure 1:**
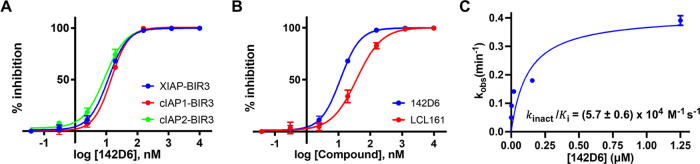
Characterization of **142D6** selectivity and its kinetics of binding to XIAP-BIR3. (A) DELFIA displacement curves relative to **142D6** against the BIR3 domains of XIAP, cIAP1, and cIAP2 with a 6 h preincubation. IC_50_ values are reported in Table 1. (B) Comparison of the activity of the reversible pan-IAP active LCL161 and **142D6** against XIAP-BIR3 in the DELFIA displacement assay with a 6 h preincubation. IC_50_ values are reported in Table 1 (IC_50_(**142D6**) = 12 ± 2 nM; IC_50_(LCL161) = 40 ± 14 nM). (C) Percent inhibitions of **142D6** at the indicated concentrations and at various time points were measured using the DELFIA assay, which was used to calculate the *k*_obs_ and the second-order rate constant *k*_inact_/*K*_i_ values (*K*_i_ = 0.1227 μM, *k*_inact_ = 0.0068 s^–1^). Data are presented as mean ± standard error (SE) of at least two replicate measurements.

Compared to the reversible ligand LCL161, IC_50_ values decrease with the preincubation period, as typical of covalent agents (Table 1). As a reference, compound **1** was also prepared and tested that is very similar to **142D6**, but it lacks the electrophile (Table 1). Subsequently, we used the DELFIA assay to determine the kinetics for compound **142D6** by monitoring displacement at different concentrations and incubation times with the BIR3 domain of XIAP (Figure 1). Kinetic values could be extrapolated in *k*_inact_/*K*_i_ = (5.7 ± 0.6) × 10^4^ M^–1^ s^–1^, which are again indicative of rapid and efficient reaction between the compound and the BIR3 domain of XIAP.

**Figure 2:**
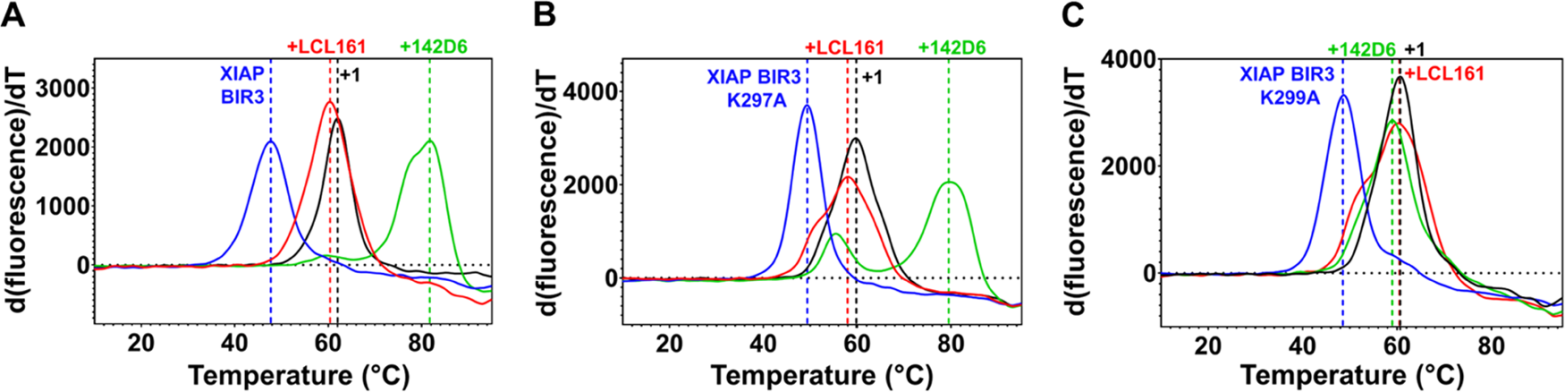
Effect of compound **142D6** on the thermal stability of XIAP-BIR3 and its mutants *in vitro*. (A) Denaturation thermal shift measurements were performed by incubating XIAP-BIR3 (10 μM) with the vehicle control (buffer with 1% DMSO) (blue), LCL161 (red, at 20 μM), **142D6** (green, at 20 μM), and **1** (black, at 20 μM), for 2 h prior to a temperature gradient (0.05 °C/s over 30 min) up to 95 °C. LCL161 induced a large denaturation thermal shift, typical of very potent low nanomolar agents. However, **142D6** induced an even greater denaturation thermal shift (Δ*T*_m_ of 34.0 ± 0.1 °C while Δ*T*_m_ for LCL161 is 12.7 ± 0.2 °C) compared to the free protein. On the contrary, eliminating the fluorosulfate as in **1**, resulted in a thermal shift that was more similar to that induced by LCL161 (Δ*T*_m_ of 14.45 ± 0.05 °C). (B) Similar measurements were also performed for the XIAP-BIR3 mutant K297A. Here, LCL161 induced Δ*T*_m_ values of 8.6 ± 0.2 °C and **142D6** induced Δ*T*_m_ values of 30.2 ± 0.1 °C. In panel (C), the measurements were performed with the XIAP-BIR mutant K299A. **142D6** induced Δ*T*_m_ values of 10.3 ± 0.2 °C that were similar to that induced by the noncovalent LCL161, or **1**, strongly indicating that the covalency observed was dependent on Lys 299. Measurements for each data point were collected in quadruplicate and the reported Δ*T*_m_ values were expressed as mean ± SE.

To further analyze XIAP-BIR3/**142D6** interactions, we measured ligand-dependent denaturation thermal shifts *in vitro*. In these experiments, the BIR3 domain of XIAP protein was incubated with vehicle control (buffer with 1% dimethyl sulfoxide (DMSO)), **LCL161**, **142D6**, or its noncovalent control (**1**), at a protein:ligand ratio of 1:2 for 2 h at room temperature (RT) in the presence of the fluorescent dye SYPRO Orange. We chose 2 h of incubation as under the ligand-to-protein ratio of 2:1, the reaction is complete within this time frame. In these measurements, samples were subjected to a gradual increase in temperature, to induce protein thermal denaturation. While the protein denatured and unfolded, nonspecific binding between the fluorescent dye and the protein’s exposed hydrophobic residues took place, that in turn sequestered the fluorescent dye from solution resulting in an increase in fluorescence. Hence, maximal fluorescence was observed at the temperature causing the protein to be fully denatured. The protein’s melting temperature (*T*_m_) is the temperature at which there is 50% denaturation. Ligand binding can stabilize or at times also destabilize the protein target to thermal denaturation, causing a significant shift in the protein denaturation temperature (Δ*T*_m_).^72,73^ In our experience with this assay, denaturation thermal shift values typically range from a fraction of 1 °C for weak binders and up to ∼10 °C for more potent inhibitors. When tested in this assay, LCL161 caused a significant stabilization of the BIR3 domain of XIAP, with a Δ*T*_m_ of 12.7 ± 0.23 typical of very potent agents.^35^ However, when **142D6** is tested in the same assay, it caused a Δ*T*_m_ of 34.0 ± 0.1 °C (Figure 2A), likely attributable to the covalent nature of the interactions. Accordingly, removal of the fluorosulfate as in compound **1** resulted in a decreased value in the denaturation thermal shift (Figure 2A). From modeling considerations, two possible XIAP-BIR3 Lys residues could react with the fluorosulfate, namely, Lys 297 and Lys 299.^39^ Hence, to investigate whether either one Lys residue was directly involved in binding covalently the BIR3 domain, ligand-induced denaturation thermal shifts were measured for the two mutants BIR3(K297A) and BIR3(K299A). The mutations had a relatively small effect on the denaturation thermal shift induced by LCL161 (Figure 2B) compared to the effect on wt-BIR3. Likewise, Δ*T*_m_ induced by **142D6** for the BIR3(K297A) (Δ*T*_m_ = 30.16 ± 0.35 °C) was somewhat comparable to that induced by wt-BIR3 (Δ*T*_m_ = 34.0 ± 0.1 °C), and still much larger than what observed with LCL161, suggesting that the covalent adduct was still forming with this mutant. On the contrary, the denaturation thermal shift was dramatically smaller in the **142D6**-treated BIR3(K299A) mutant (Δ*T*_m_ = 10.3 ± 0.2 °C) and more aligned with a noncovalent binding (Figure 2C). Peak symmetry was observed for unbound proteins in all mutants while some asymmetry can be observed in the denaturation curves of compound-bound forms. This may be due to either complete saturation of protein by the ligands. For example, this can be seen in panel B with agent **142D6**. Mutation of the adjacent Lys297 may reduce the nucleophilicity of the targeted Lys 299. It is also possible that the ligand-bound protein exhibits a different denaturation pathway as observed with ligand LCL161 in panels B and C. All in all, these data pointed to XIAP K299 as the covalently modified residue.

**Figure 3:**
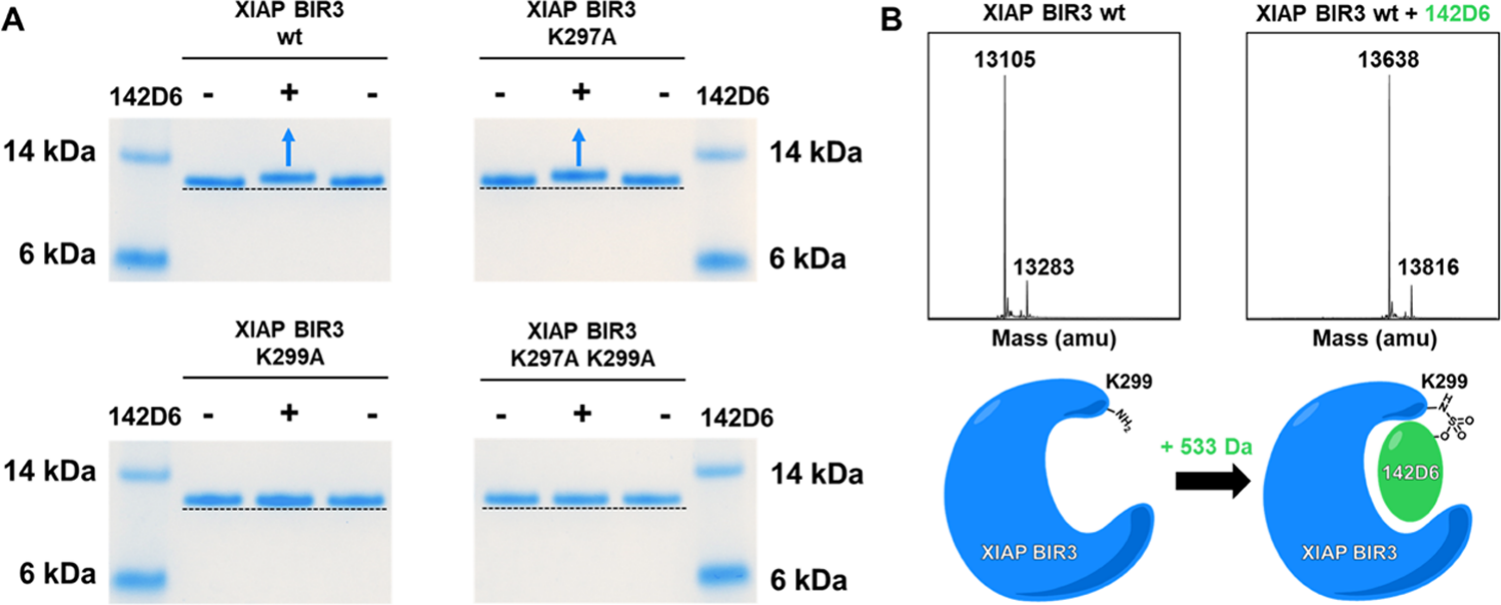
**142D6** targets XIAP-BIR3 residue Lys 299. (A) SDS-polyacrylamide gel electrophoresis (PAGE) followed by Coomassie staining of the BIR3 domain of XIAP wild-type, Lys297Ala, Lys299Ala, and Lys297Ala/Lys299Ala. All proteins were incubated at 10 μM for 2 h at 25 °C in the presence and absence of 20 μM **142D6** in 25 mM Tris pH 8, 150 mM NaCl, 1 mM dithiothreitol (DTT), and 50 μM Zn(Ac)_2_. (B) MS spectra of the BIR3 domain of XIAP collected in the absence (left) and in the presence of **142D6**. The mass increase of the BIR3 domain by 533 Da is observed with wt-BIR3 and with the BIR3(K297A) but not or in a very small amount with the BIR3(K299A) mutant (not shown).

The covalent adduct formation between **142D6** and the BIR3 domain of XIAP and the K297A mutant was further verified by sodium dodecyl sulfate (SDS) gel electrophoresis and mass spectrometry data (Figure 3), whereas no adduct formation was observed in the BIR3(K299A).

**Figure 4:**
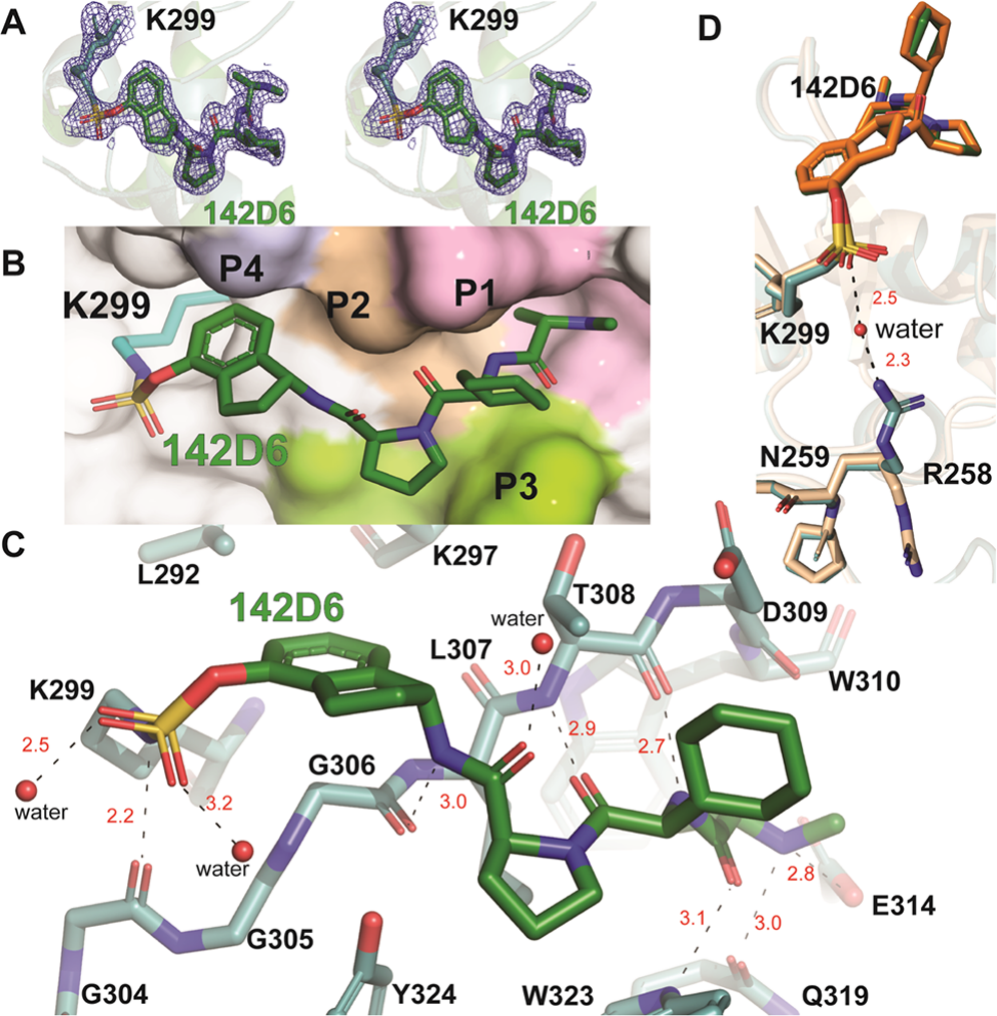
Characterization of XIAP-BIR3 in complex with **142D6** (PDB ID 8GH7). (A) Wall-eyed stereo view of **142D6** (green) covalently linked to XIAP-BIR3. Blue mesh is the 2 *F*_o_ – *F*_c_ density rendered at 1σ from a composite omit map. (B) Location of **142D6** into the BIR3 binding pocket. The position of K299 is rendered cyan in stick form. The surface of XIAP-BIR3’s P1–P4 binding positions are colored, respectively, as light pink, wheat, limon, and blue-white. (C) Molecular interactions of **142D6** within the BIR3 binding site. Waters are rendered as red spheres. The black dashes represent hydrogen bonds, while distances (Å) are in red. (D) Overlay of monomer A (teal) and B (wheat) from the crystal structure of the XIAP BIR3–**142D6** complex. Water molecules and distances related to R258s from monomer A with **142D6** (forest) are labeled as in (C). **142D6** belonging to monomer B is shown in orange.

To further characterize the binding of **142D6** with XIAP, we solved the X-ray structure of the complex between the agent and the BIR3 domain of XIAP. **142D6** covalently bound to XIAP-BIR3 crystallized under several conditions. A data set with a resolution of 1.75 Å (Table S1) was obtained from crystals derived by protein covalently modified by **142D6**. Globally, the XIAP-BIR3/**142D6** resembled other previous XIAP-BIR3 structures in both secondary and tertiary structures. Molecular replacement readily obtained a solution in the space group *P*2_1_2_1_2_1_ using XIAP-BIR3 (PDB 5C0K) as a search model.^74^ Closer inspection of both monomers in the asymmetric unit revealed continuous *F*_o_ – *F*_c_ density with XIAP Lys 299 within the BIR3 domain (Figure S1). Prior to and after refinement, this density was an exact fit for **142D6** (Figure 4A). This placed the **142D6** adduct within the P1–P4 binding sites of XIAP-BIR3, a placement that was driven by several of electrostatic and hydrophobic interactions as highlighted in Figure 4B,C. In particular, within the P1 site, **142D6**’s methyl group points in a pocket formed by Leu307 and Trp310 (Figure 4C). The *N*-methyl-l-alanine of **142D6** forms a hydrogen bond network with the sidechains of Glu314, Gln319, and Trp323, which span across the P1 and P3 interfaces (Figure 4C). Engagement with the P2 site is observed to be primarily driven by a β sheet like hydrogen bonding network that pairs up the carbonyl and amine of **142D6**’s l-cyclohexyl glycine with the corresponding main-chain amine of Leu307 and carbonyl Thr308 (Figure 4C). This leaves the cyclohexyl sidechain pointing out toward the bulk solvent in a manner that takes advantage of a hydrophobic surface by the P3 residues Trp323 and Leu344 (Figure 4C). The proline moiety of **142D6** also interacts with this same hydrophobic cluster. Interactions between **142D6** and residues surrounding P4 occur via a hydrogen bond with Gly306 and the amine from 1-aminoindane of **142D6** with the sidechain of this moiety inserting itself into a pocket formed by the sidechains of Lys297 and Leu292 along with a portion of the main chain in XIAP-BIR3 (Figure 4C). Apart from the covalent interaction formed between the **142D6** sulfate and Lys 299, the orientation of the sulfamate is also stabilized by a hydrogen bond with the carbonyl of Gly304 (Figure 4C). While this interaction is the sole noncovalent interaction observed in monomer B of the crystal structure, Arg258 in the monomer A structure forms a water-mediated hydrogen bonding network (Figure 4D). These interactions are similar to those observed in other ligands bound to the BIR3 domains of IAPs.^45,46,50,52,58,64^

### Cellular and *In Vivo* Pharmacology Studies

As we previously reported,^39^**142D6** is long-lived (several hours) and soluble up to 2 mM in aqueous buffer (pH = 7.5, *T* = 25 °C; Figure S3), similar to what we observed with LCL161. Likewise, we observed that **142D6** presented a similar plasma stability (*t*_1/2_ > 2 h) as LCL161.^39^ We also reported that the compound is cell permeable by western blot analysis using a cell line that we stably transfected with HA–BIR3 of XIAP.^75^

**Figure 5:**
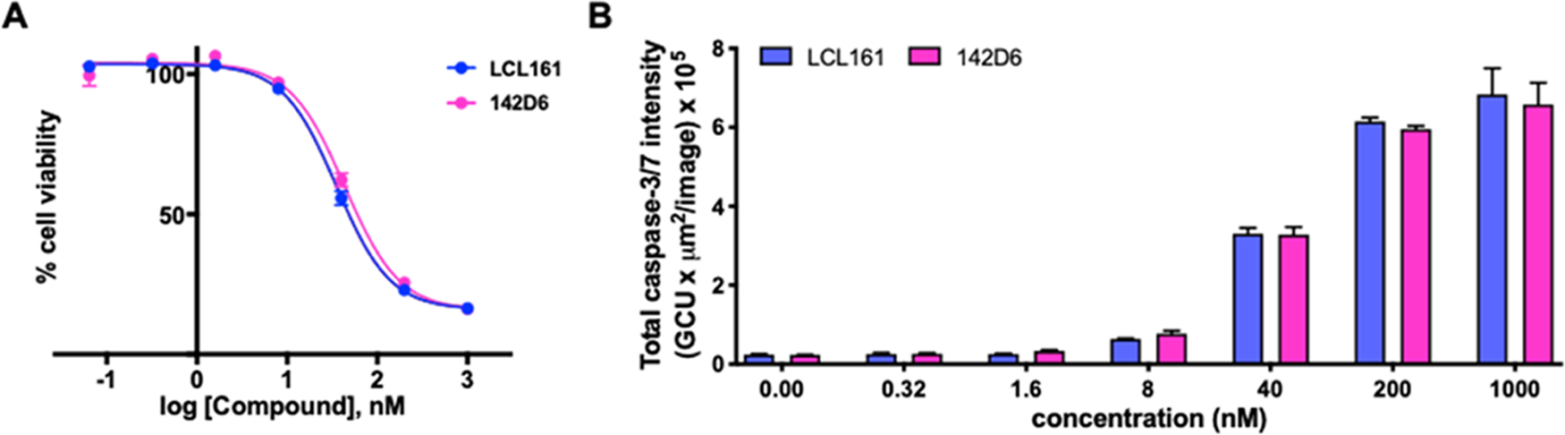
Cell viability and apoptosis assays. MDA-MB-231 NucLight Red cells were treated with different concentrations of compounds and red fluorescence was detected at various time points with the IncuCyte S3 live-cell analysis system. (A) Dose–response curves (blue, LCL161; pink, **142D6**) are reported for the 72 h time point. (B) Histograms displaying caspase-3/7 activity of MDA-MB-231 NucLight Red cells measured at the 24 h time point. Data are presented as mean ± SE of at least two independent experiments.

Here, we further compared the pharmacological properties of the agent side by side with LCL161 against the breast cancer cell line MDA-MB-231 in 2D and 3D cultures. First, we monitored cell viability using a live-cell analysis via the IncuCyte S3 (Sartorius) system. For this purpose, we obtained MDA-MB-231 cells that were labeled with IncuCyte NucLight Reagents. This approach facilitated efficient nuclear labeling of MDA-MB-231 cells, using a lentiviral-based labeling reagent that enabled the expression of a nuclear-restricted red (mKate2) fluorescent protein. Hence, cell viability was monitored in the presence and absence of various doses of **142D6** or LCL161 and red fluorescence was monitored over time (0–72 h) (Figure 5A). Subsequently, induction of apoptosis was further examined in MDA-MB-231 NucLight Red cells via the IncuCyte S3 caspase-3/7 green fluorescence-based apoptosis assay. In this assay, cells were exposed to agents and to a nonfluorescent caspase-3/7 substrate that passively crossed the cell membrane. Once inside the cell, the substrate was cleaved by activated caspase-3/7, resulting in a release of a green DNA-binding fluorescent dye. Therefore, after exposure to various doses of **142D6** or LCL161, apoptotic cells could be identified by the appearance of fluorescently labeled nuclei (Figure 5B).

Both assays revealed that **142D6** presented a similar potency to the clinical candidate LCL161 (Figure 5) with the EC_50_ values of 44 ± 4 and 37 ± 5 nM in the cell viability assays, respectively, and with EC_50_ values of 61 ± 12 and 57 ± 12 nM in the apoptosis assays, respectively.

**Figure 6:**
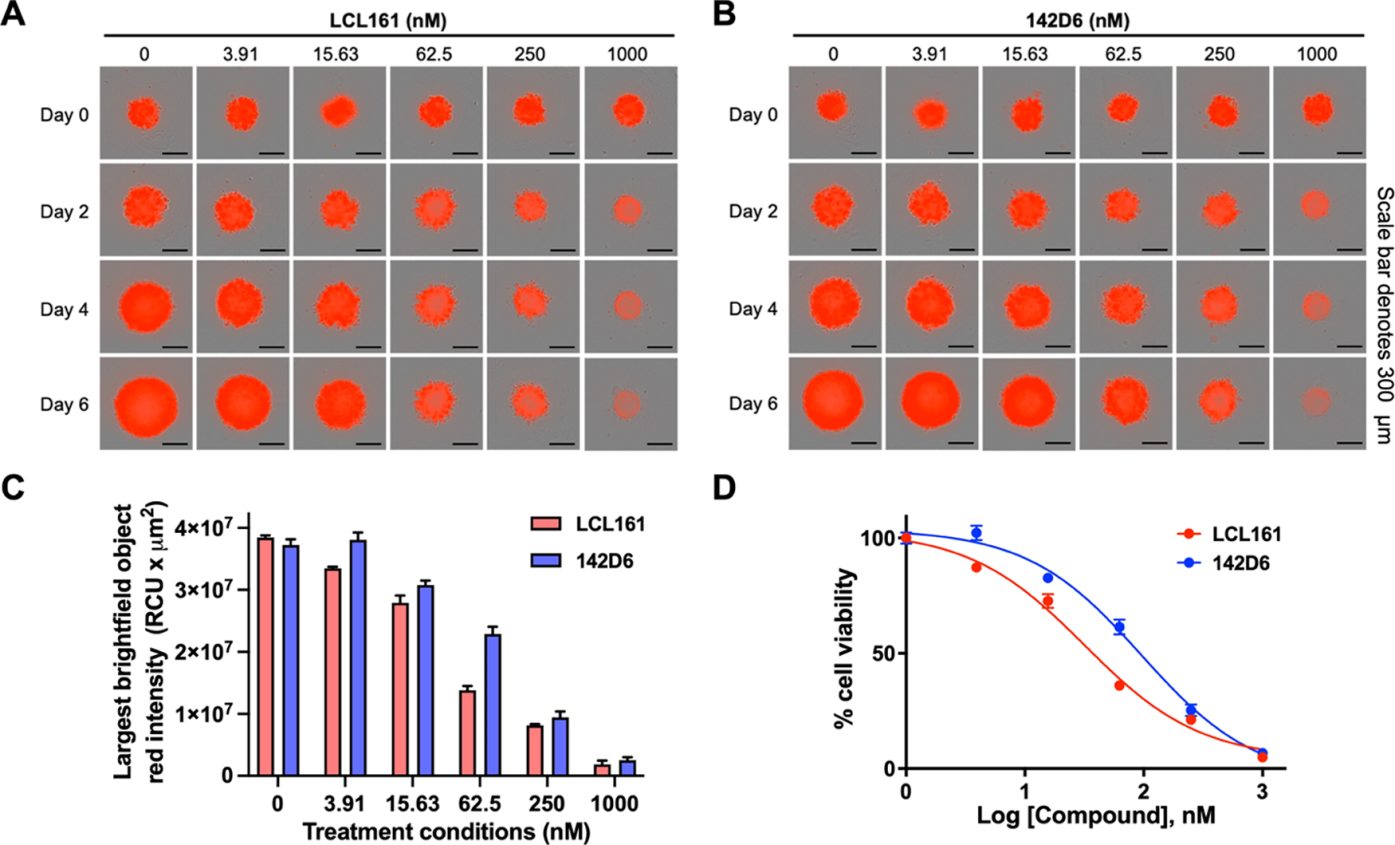
Activity of LCL161 and **142D6** in the 3D cell culture with MDA-MB-231 NucLight Red cells. Briefly, cells were seeded into 96-well round-bottom, ultralow attachment plate with the addition of Matrigel and left undisturbed for 3 days. Cells were then treated with (A) LCL161 or (B) **142D6** at the indicated doses and imaged every 6 h for 6 days with the IncuCyte live-cell analysis system. (C) Histogram showing the red fluorescence intensity of cells treated with LCL161 or **142D6** at day 6 measured by the integrated IncuCyte S3 spheroid software. (D) EC_50_ values based on the red fluorescence intensity at day 6 for LCL161 and **142D6** were determined to be 32.5 ± 5.1 and 94.2 ± 6.2 nM, respectively.

To further compare the tumor penetration ability of the agents, we monitored their cell-killing ability in 3D cultures with MDA-MB-231 NucLight Red cells. Cells were plated in 96-well round-bottom, ultralow attachment plates and left undisturbed for 3 days. Once spheroids formed, they were treated with LCL161 or **142D6** for 6 days with images taken every 6 h. The red fluorescence intensity was quantified by the integrated IncuCyte S3 spheroid software, which was then used to calculate EC_50_ values. LCL161 and **142D6** displayed an EC_50_ value of 32.5 ± 5.1 and 94.2 ± 6.2 nM, respectively (Figure 6).

**Figure 7:**
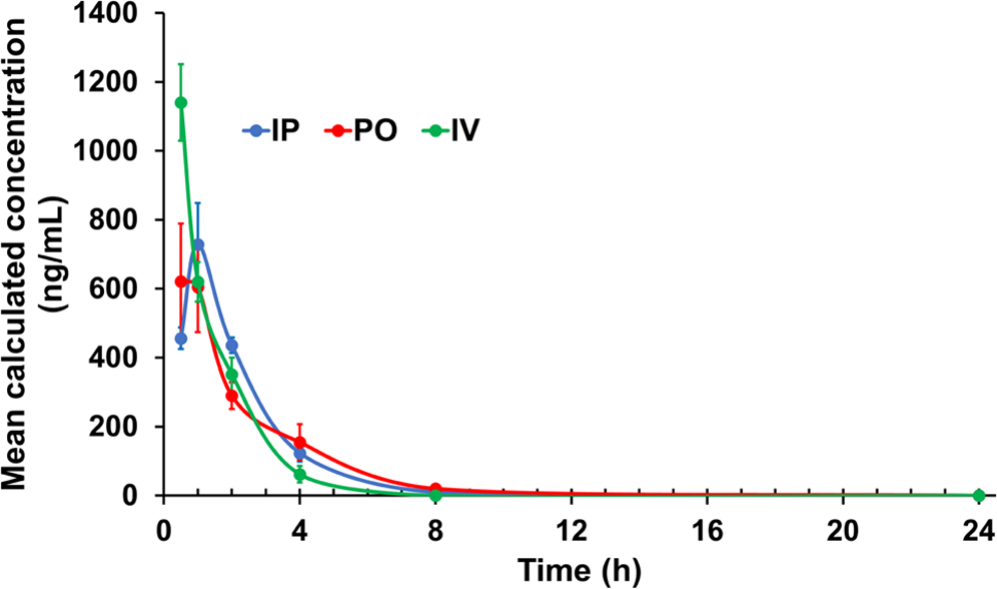
*In vivo* pharmacokinetics data for **142D6**. Balb/c mice (*n* = 5) received **142D6** in a formulation of 80:10:10 (PBS/ethanol/Tween80) in the doses of 30 mg/kg (PO and IP) or 10 mg/kg (IV) and drug plasma concentration was measured at times 30 min, 1 h, 2 h, 4 h, 8 h, and 24 h.

While these data demonstrate that aryl-fluorosulfate can be a viable pharmacological tool, its translation to possible therapeutics requires more stringent pharmacokinetic properties. Hence, to further probe the pharmacokinetic properties of **142D6**, we tested it in mice after administration of the agent via the oral (PO), intravenous (IV), or intraperitoneal (IP) routes and measured drug plasma concentration at various times. Balb/c mice (*n* = 5 per group) received **142D6** at 30 mg/kg IP, PO, and at 10 mg/kg IV, and drug plasma concentration was measured via liquid chromatography-mass spectrometry (LC/MS) 30 min, 1 h, 2 h, 4 h, 8 h, and 24 h after administration (Figure 7). The data show that in each case, the compound reaches plasma concentrations well above the cellular 2D or 3D EC_50_ values. Perhaps, most importantly, the agent is orally bioavailable with a calculated bioavailability for the oral route, *F*_PO_, of 36%. For practical purposes, the IP bioavailability is also relatively good (*F*_IP_ 35%), which should facilitate the use of **142D6** in mouse models of cancer that are driven by IAPs’ expressions.

## Conclusions

Recent manuscripts have reported on the possibility of covalent targeting of Lys residues in active sites of proteins by the introduction of appropriately placed electrophiles on existing ligands.^26,27,29,76^ Our own recent studies^24,25,39^ and others^26,28,77−79^ also revealed that it is possible to target Lys residues located at protein–protein interfaces, suggesting that in principle, the target space of covalent antagonists of PPIs could be expanded to include Lys. To be useful as pharmacological tools or even therapeutics, these electrophiles need to possess a proper balance between stability and selectivity and react only with the intended residue. Our data answer common questions when proposing novel electrophiles in covalent ligand design. First, structural studies on the complex revealed at high resolution the geometry of the sulfamate bond between the reactive fluorosulfate and Lys 299. Moreover, **142D6** was chemically stable in buffer and plasma and reacted covalently in a very selective way. Indeed, exposure of the BIR3(K299A) protein, containing 6 Lys, 6 Tyr, and 11 His residues, to **142D6** did not result in a covalent adduct as assessed by SDS electrophoresis, denaturation thermal shift, and mass spectrometry analyses. The agent was effective as the clinical candidate LCL161 in 2D and 3D culture against breast cancer MDA-MB-231 cells and, perhaps most importantly, it was found to be orally bioavailable in pharmacokinetics studies in mice. These observations should once again corroborate our observations that aryl-fluorosulfates should be given serious consideration in widening the target space from the Cysteinome^15−17^ to other more abundant residues such as Lys,^80,81^ as shown here, or also Tyr, or His as we and others demonstrated recently.^25,36,82^ In particular, our studies should further encourage considering the incorporation of aryl-fluorosulfates, when possible, within drug discovery strategies that include structure-based approaches, but also in unbiased screening campaigns including NMR-based approaches,^66,83−91^ or DNA-encoded libraries.^81,92−94^ Based on these considerations, we are confident that aryl-fluorosulfates could find broader applications in the design of chemical probes, pharmacological tools, or even therapeutics.

## Experimental Section

### General Chemistry

For the synthesis of **142D6** and **1**, we used solvent and reagents commercially available, and used without further purification. The correct concentration of the agents was verified by NMR spectroscopy on a Bruker Avance III 700 MHz instrument. High-resolution mass spectral data were acquired on an Agilent LC-TOF instrument. The compounds were purified by reversed-phase high-performance liquid chromatography (RP-HPLC) on a JASCO preparative system equipped with a PDA detector and a fraction collector controlled by a ChromNAV system (JASCO) on an XTerra C18 10 μm 10 mm × 250 mm (Waters) to >95% purity. LCL161 was obtained from MedChem Express. BAL resin was purchased from Creosalus. Fmoc-amino acids were purchased from Chem-Impex and Novabiochem. The [4-(acetylamino)phenyl]imidodisulfuryl difluoride (AISF) reagent was purchased from Sigma-Aldrich. The synthesis was performed in-house by standard solid-phase Fmoc peptide synthesis protocols on BAL resin. For each coupling reaction, 3 equiv of Fmoc-AA, 3 equiv of 1-[bis(dimethylamino)methylene]-1*H*-1,2,3-triazolo[4,5-*b*]pyridinium 3-oxid hexafluorophosphate (HATU), and 5 equiv of *N*,*N*-diisopropylethylamine (DIPEA) in 1 mL of dimethylformamide (DMF) were used. The coupling reaction was allowed to proceed for 1 h at room temperature, followed by three washes with DMF. Kaiser test was employed to monitor reaction completion. Fmoc deprotection was performed in two steps by treating the resin-bound peptide with 20% 4-methylpiperidine in DMF for 5 min and then 15 min at room temperature. The purity of tested compounds was assessed by HPLC using an Atlantis T3 3 μm 4.6 × 150 mm^2^ column (H_2_O/acetonitrile gradient from 5 to 100% in 45 min). Agents have a purity of >95% (Figure S2).

#### Compound **142D6**

The synthesis of (*R*)-1-((*S*)-1-((*S*)-2-cyclohexyl-2-((*S*)-2-(methylamino)propanamido)acetyl)pyrrolidine-2-carboxamido)-2,3-dihydro-1*H*-inden-4-yl sulfurofluoridate was reported previously by us.^39^ Briefly, BAL resin (0.05 mmol scale) was loaded using a solution of (*R*)-1-amino-indan-4-ol (3 equiv) in DMF and shaken for 30 min, followed by reduction using sodium triacetoxyborohydride (3 equiv, overnight at RT). The resin was subsequently filtered and washed three times with DMF, three times with dichloromethane (DCM) (3×), and again three times with DMF. For the coupling of Fmoc-proline on the secondary amine, the reaction time was increased to 2 h. Fmoc deprotection and peptide elongation then followed standard procedures described in the General Chemistry section. Aryl-fluorosulfate incorporation was performed on resin, using the [4-(acetylamino)phenyl]imidodisulfuryl difluoride (AISF)^37^ reagent (1.2 equiv, 2.2 equiv of 1,8-diazabicyclo[5.4.0]undec-7-ene (DBU) in tetrahydrofuran (THF), overnight reaction at room temperature). After cleavage, the crude was purified by preparative RP-HPLC and water/acetonitrile gradient (5–100%) containing 0.1% trifluoroacetic acid (TFA). High-resolution mass spectrometry (HRMS): calcd 552.2418 (M); obs 553.4656 (M + H)^+^.

#### Compound **1**

The synthesis of (*S*)-1-((*S*)-2-cyclohexyl-2-((*S*)-2-(methylamino)propanamido)acetyl)-*N*-((*R*)-4-hydroxy-2,3-dihydro-1*H*-inden-1-yl)pyrrolidine-2-carboxamide was performed in the same way described for **142D6**, without the incorporation of the aryl-fluorosulfate warhead. HRMS: calcd 470.2893 (M); calcd 470.2893 (M); obs 471.2976 (M + H)^+^, 493.2785 (M + Na)^+^.

### Protein Constructs, Expression, and Purification

cDNA fragments encoding the human BIR3 domain of XIAP (residues 253–347 for wt protein and mutants K297A, K299A, and K297A/K299A) and an N-terminal His tag were subcloned into a pET15b vector.^24^ The plasmids were transformed into *E. coli* BL21-Gold(DE3) pLysS cells and grown in Luria–Bertani (LB) medium at 37 °C with 100 μg/mL of ampicillin until reaching an OD_600_ of 0.6–0.7, followed by induction with 1 mM isopropyl β-d-1-thiogalactopyranoside (IPTG) overnight at 25 °C. Bacteria were then collected by centrifugation and lysed by sonication at 4 °C. Proteins were purified using Ni^2+^ affinity chromatography, eluted in 25 mM Tris at pH 7.5, 500 mM NaCl, and 500 mM imidazole, and exchanged and further purified with a size-exclusion chromatography column (HiLoad 26/60 Superdex 75 prep grade) into an aqueous buffer composed of 25 mM Tris at pH 8, 150 mM NaCl, 50 μM Zn(Ac)_2_, and 1 mM DTT.

### Dissociation-Enhanced Lanthanide Fluorescence Immunoassay (DELFIA)

The biochemical assay we used to quantify the displacement by test agents of the reference tetrapeptide AVPI from the BIR3 domains of XIAP, cIAP1, and cIAP2 was described by us previously. Briefly, 100 μL of 100 nM AVPI-Biotin (AVPIAQKSEK-Biotin) was added to each well of the 96-well streptavidin-coated plates (PerkinElmer), incubated for 1 h, followed by three washing steps to remove the unbound AVPI-Biotin. A solution containing 89 μL of Eu-N1-labeled anti-6x-His antibody (PerkinElmer) was subsequently added, followed by 11 μL of a mixture containing the protein domain and a serial dilution of the test compounds. These were incubated for 2 h with or without preincubation. Preincubation consisted of a 6 h incubation period for only the protein and the test agents. After washing steps with DELFIA wash buffer (PerkinElmer), 200 μL of the DELFIA enhancement solution (PerkinElmer) was added to each well, and fluorescence was measured using a VICTOR X5 microplate reader (PerkinElmer; excitation and emission wavelengths of 340 and 615 nm). For these assays, the protein concentrations used were 30 nM for XIAP-BIR3 and cIAP1-BIR3, and 15 nM for cIAP2-BIR3. The antibody concentrations in a solution of 89 μL used for XIAP-BIR3 and cIAP1-BIR3 were 1:2000 and 1:1500, respectively, for cIAP2-BIR3. Protein, peptide, and antibody solutions were prepared with DELFIA assay buffer (PerkinElmer) and all of the incubations were performed at room temperature. Samples were normalized to 1% DMSO and reported as % inhibition. The IC_50_ values were calculated from dose–response curves using GraphPad Prism version 9. The reported SE values were obtained from replicate measurements.

Kinetic measurements were performed in the same manner as described above for the incubation and washing steps of streptavidin-coated plates and the biotinylated peptide. Each well was subsequently incubated with 30 nM XIAP-BIR3 and 1:2000 Eu-N1-labeled anti-6x-His antibody for 2 h prior to incubation with **142D6** for 0, 2, 5, 10, 20, and 40 min. Plates were washed three times and incubated with 200 μL of enhancement solution for 10 min. Fluorescence measurements were taken as described previously. The slope of a percent inhibition *versus* incubation time plot was used to calculate the observed rate constant for inhibition, *k*_obs_, which was then replotted against the peptide concentrations and fitted to a hyperbolic curve on Prism 9 (GraphPad) to extrapolate the inhibition constant, *K*_i_, and *k*_inact_.

### Gel Electrophoresis

Each protein was incubated at a concentration of 10 μM with and without 20 μM **142D6** for 2 h at room temperature in a buffer containing 25 mM Tris pH 8, 150 mM NaCl, and 50 μM zinc acetate. Samples were loaded onto the NuPAGE 12% Bis–Tris protein gels (Life Technologies) and electrophoresed using MES running buffer (Life Technologies) at 200 V for 35 min. Gels were then stained with SimplyBlue SafeStain (Life Technologies) according to the manufacturer’s protocol.

### Cell Culture and Nuclear Labeling

MDA-MB-231 breast cancer cell line was purchased from the American Type Culture Collection (ATCC). Cells were then nuclear-labeled red (MDA-MB-231 NucLight Red cells) with the IncuCyte NucLight red lentivirus reagent (Sartorius) according to the manufacturer’s protocol. MDA-MB-231 NucLight Red cells were cultured in Dulbecco’s modified Eagle’s medium (DMEM; Corning) supplemented with 10% fetal bovine serum (FBS; Gibco) and 1 μg/mL puromycin and maintained in a humidified incubator at 37 °C with 5% CO_2_.

### Caspase-3/7 Assay, Cell Viability Assay, and 3D Culture

MBD-MB-231 NucLight Red cells were seeded at 10,000 cells/well in 96-well plates and allowed to attach overnight. The media were removed, and cells were treated with various concentrations of LCL161 or **142D6** in the presence of 2.5 μM of the IncuCyte Caspase-3/7 Green Apoptosis Reagent (Sartorius) for 4 days. Live images were taken every 3 h with the IncuCyte S3 live-cell analysis system and the data at the 24 h time point were analyzed. Cell viability was concurrently measured along with the caspase-3/7 assay by quantifying the number of red fluorescent nuclei from each treatment and normalized to those of the DMSO-treated wells at 72 h. The Top-Hat method was used to subtract background noise from the red and green channels.

To create 3D spheroids, 2500 MDA-MB-231 NucLight Red cells were plated into 96-well round-bottom, ultralow attachment plates (Corning, Cat# 7007) with the addition of 2.5% Matrigel for 3 days until spheroids reached approximately 350 μm in size. Spheroids were then treated with the indicated concentrations of LCL161 or **142D6** in the presence of 0.1% DMSO and imaged with the IncuCyte S3 live-cell analysis system every 6 h for 6 days. The integrated IncuCyte S3 spheroid software was used to analyze the red fluorescence intensity.

### Crystallization of XIAP-BIR3/**142D6**

The XIAP-BIR3/**142D6** covalent adduct complex was screened against a range of QIAGEN NeXtal suite conditions by the hanging drop format using a TTP Labtech Mosquito (TTP Labtech, Hertfordshire, U.K.). In short, XIAP-BIR3/**142D6** crystals were grown from 15 mg/mL purified protein in 25 mM *N*-(2-hydroxyethyl)piperazine-*N*′-ethanesulfonic acid (HEPES) (pH 7.6), 50 mM NaCl, 1 mM MgSO_2_, and 0.25 mM tris(2-carboxyethyl)phosphine (TCEP) buffer solution by hanging drop vapor diffusion at 20 °C against 500 μL of crystallization precipitant. Utilizing an initial hit poly(ethylene glycol) (PEG) II Suite well number 53 that contained 0.1 M HEPES pH 7.5 and precipitant 10% (w/v) PEG 4000, 20% (w/v) isopropanol crystals were further optimized yielding the final crystal conditions to be 35% isopropanol and 18% PEG 4000. With mother liquor serving as a suitable cryo, crystals were mounted and flash-cooled in liquid nitrogen flash prior to data collection. Data sets were collected at an Advanced Photon Source (Argonne National Labs, Argonne, IL) on SBC-CAT beamline ID-19 using a PILATUS3 X 6M detector. Data were collected using a wavelength of 0.97934 Å (Table S1).

### Data Processing and Structure Solutions

X-ray images were indexed, integrated, and scaled in the *P*2_1_2_1_2_1_ space group using the HKL2000 suite.^95^ Cross-validation and initial data analysis were performed using the Phenix suite of programs.^96^ The initial-phase solutions were established using molecular replacement via Phaser using 5C0K as a search model.^97^ AutoBuild iterative cycles of protein and water model building with COOT 0.7.1 and refinement with Phenix were performed.^98^ Subsequently, PEG and other ligands were individually placed into structures based on *F*_o_ – *F*_c_ density at 3σ and refined with Phenix yielding a fitting 2 *F*_o_ – *F*_c_ density at 1σ for the ligands.^99^ The final model of each structure was examined via Molprobity to confirm the quality of the structures.^100^ The data collection and refinement statistics for each structure are listed in Table S1.

### Denaturation Thermal Shift Assays

Thermal shift assays for BIR3/BIR3 K297A/BIR3 K299A construct/inhibitor complexes were obtained with a BioRad CFX Connect Real-Time PCR Detection System. Each data point was collected in quadruplicate. Incubation of the BIR3 protein or mutants with compounds was performed at 25 °C for 2 h. Protein/compound complexes and 5000× SYPRO Orange dye (Sigma) were diluted using reaction buffer, 50 mM Tris pH 8.0, 150 mM NaCl, 50 μM zinc acetate, to obtain final concentrations of 10 μM protein, 20 μM compound, and 10× SYPRO Orange. Sample plates were heated from 10 to 95 °C with heating increments of 0.05 °C, over 30 min. Fluorescence intensity was measured within the excitation/emission ranges 470–505/540–700 nm.

### *In Vivo* Pharmacokinetics

*In vivo* pharmacokinetics studies were conducted at the University of California San Diego Pharmacology core facility, according to IACUC-approved protocols. **142D6** was dissolved in a formulation composed of 80% PBS, 10% ethanol, and 10% Tween80. In this formulation, the agent was soluble at 7.5 mg/mL and used for the oral (PO) and intraperitoneal (IP) administrations (∼100 mM, adjusted for body weight, of 7.5 mg/kg to give 30 mg/kg). A similar solution was prepared containing 2.5 mg/mL of **142D6**, and it was used for the intravenous (IV) dose (∼100 mM, adjusted for body weight, of 2.5 mg/kg to give a dose of 10 mg/kg, to each or five mice). Five mines per treatment group were used and blood was drawn at times 30 min, 1 h, 2 h, 4 h, 8 h, and 24 h. Drug plasma concentration was determined by an LC/MS method that was previously calibrated on a known amount of **142D6**.

## Supporting Information Available

Crystallographic parameters (Table S1); time-dependent cell viability data for two cell lines and each of the compounds reported in Table 1 (Figure S1); the HPLC trace for the **142D6** batch used for current experiments (Figure S2); and chemical stability data for **142D6** using 1D ^1^H NMR (Figure S3) (PDF)
Molecular formula strings file (CSV)

## Accession Codes

The atomic coordinates and structure factors have been deposited in the RCSB Protein Data Bank, www.rcsb.org, PDB ID 8GH7. The PDB DOI for this deposition is 10.2210/pdb8gh7/pdb. The authors will release the atomic coordinates upon article publication.

## Notes

**142D6** has been patented by the University of California, Riverside (UCR). M.P., C.B., and P.U. are listed as co-inventors in the issued patent and may receive a share of eventual future royalties if the agent gets licensed, according to the University of California policies. M.P. is co-founder of Armida Labs., Inc. (Riverside) that may license **142D6** from UCR.

## Abbreviations Used

XIAP: X-linked inhibitor of apoptosis protein
BIR: baculovirus IAP repeat domains
cIAP1: cellular inhibitor of apoptosis protein 1
cIAP2: cellular inhibitor of apoptosis protein 2
DELFIA: dissociation-enhanced lanthanide fluorescent immunoassay
DMF: dimethylformamide
HATU: 1-[bis(dimethylamino)methylene]-1*H*-1,2,3-triazolo[4,5-*b*]pyridinium 3-oxid hexafluorophosphate
DIPEA: *N*,*N*-diisopropylethylamine
AISF: [4-(acetylamino)phenyl]imidodisulfuryl difluoride
DBU: 1,8-diazabicyclo[5.4.0]undec-7-ene
THF: tetrahydrofuran
