# Supplementary material for: Characterization of a Potent and Orally Bioavailable Lys-Covalent Inhibitor of Apoptosis Protein (IAP) Antagonist
Source: J Med Chem. 2023 Jun 1;66(12):8159–69. doi: 10.1021/acs.jmedchem.3c00467 (PMC10291551; doi:10.1021/acs.jmedchem.3c00467)

Supplemental Information

Characterization of a potent and orally bioavailable Lys-covalent Inhibitor of Apoptosis Proteins (IAP) Antagonist

Parima Udompholkul[1], Ana Garza-Granados[1], Giulia Alboreggia,[1] Carlo Baggio[1], Jack McGuire[1], Scott D. Pegan[1], and Maurizio Pellecchia [1,]*

[1]*Division of Biomedical Sciences, School of Medicine, University of California Riverside, 900 University Avenue, Riverside, CA 92521, USA.*

Table S1 page S2

XIAP BIR3 142D6 complex X-ray Data collection and Refinement Statistics

Figure S1 page S3

Wall-eyed stereo view of 142D6 (green) covalently linked to XIAP-BIR3. Blue mesh is the 2 F_o-F_c density rendered at 3.0 σ from a composite omit map.

Figure S2 page S4

HPLC trace for 142D6

Figure S3 page S5

Chemical stability data for 142D6\

*Corresponding author: Maurizio Pellecchia, phone number: (951) 827-7829; email address: maurizio.pellecchia@ucr.edu

Table S1. XIAP BIR3 142D6 complex X-ray Data collection and Refinement Statistics (Molecular replacement)

XIAP BIR3 -142D6	
**Data collection**	
Space group	$P2_12_12_1$
Cell dimensions	
a, b, c, (Å)	34.2, 55.2,
α , β, γ (°)	93.0
Wavelength	90, 90, 90
Resolution (Å)	0.97
	50.0-1.75
R_{pim} (%)	(1.78-1.75)
R_{merge} (%)	59.0 (30.5)
$I / \sigma I$	15.8 (76.3)
$CC_{1/2}$	13.37 (1.54)
Completeness (%)	0.972 (0.846)
Redundancy	99.2 (98.9)
	6.6 (5.7)
**Refinement**	
Resolution (Å)	
No. reflections	32.08 – 1.75
R_{work} (%) / R_{free} (%)	18274 (1771)
No. atoms	17.7 / 20.9
Protein	
Ligand	1511
Water	108
B factors	90
Protein	
Ligand	31.10
Water	39.09
R.m.s. deviations	41.0
Bond lengths (Å)	0.005
Bond angles (°)	0.71
Ramachandran favored (%)	98.35
Ramachandran allowed (%)	1.65
Ramachandran outliers (%)	0.00

[a] Values in parentheses are for highest-resolution shell. The dataset was collected from a single crystal.

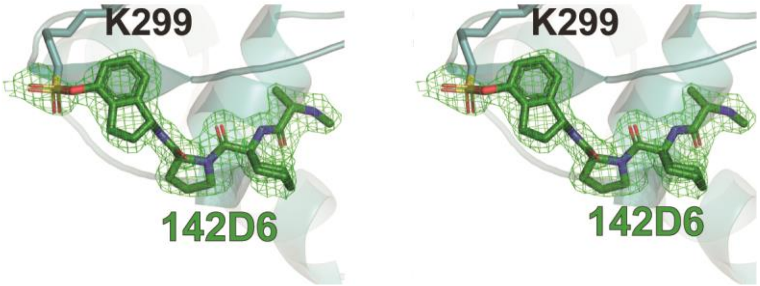

Figure S1. Wall-eyed stereo view of 142D6 (green) covalently linked to XIAP-BIR3. Blue mesh is the 2 F_o-F_c density rendered at 3.0 σ from a composite omit map.

Figure S2. HPLC trace for 142D6 (purity > 99%).

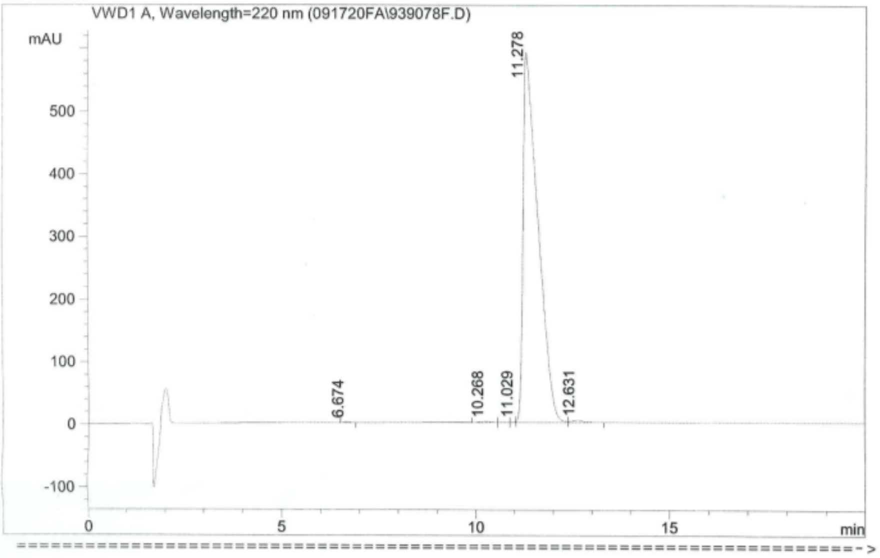

Signal 1:VWD1 A, Wavelength=220 nm

Peak #	RT [min]	Type	Height	Width [min]	Area	Area %
1	6.674	BBA	1.162	0.126	9.388	0.059
2	10.268	BV	1.791	0.219	25.605	0.160
3	10.721	VP	0.321	0.157	3.188	0.020
4	11.029	VV	1.831	0.032	3.516	0.022
5	11.278	VV	587.536	0.394	15879.340	99.241
6	12.631	VBA	3.932	0.298	79.732	0.498

Figure S3. Chemical stability of **142D6** in 25 mM TRIS pH = 8, 150 mM NaCl. 1D ^{1}H NMR spectra of the agent were collected at different time points at 25 ^{o}C, and compared. No appreciable differences can be noted at any of the data points collected, indicating a stable agent under these experimental conditions.

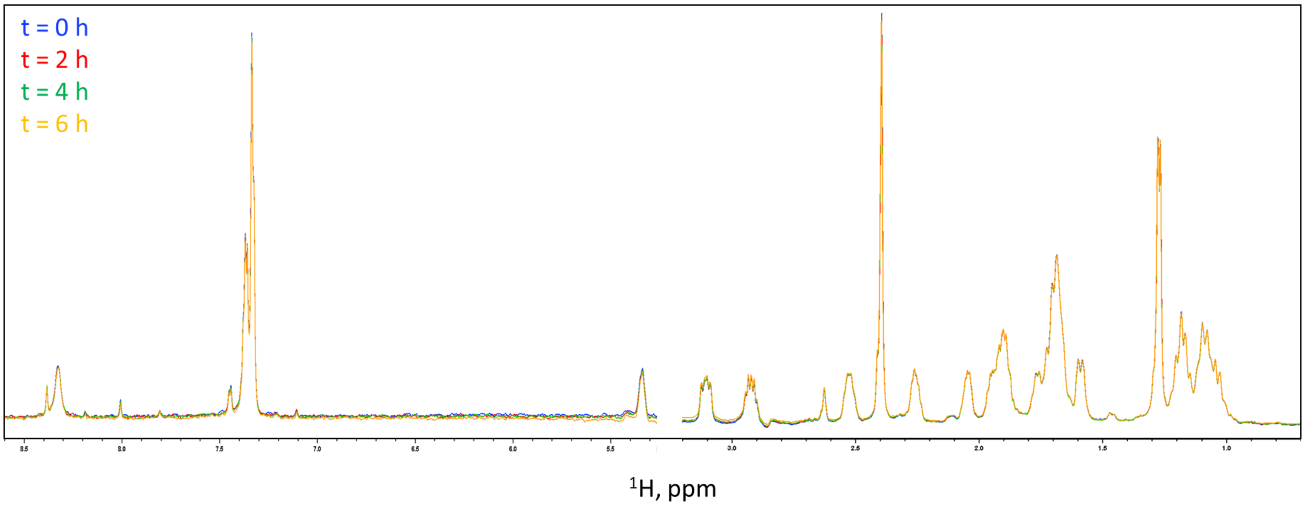

Supplement: Supplementary file 1 — jm3c00467_si_001.pdf [file jm3c00467_si_001.pdf]
